# Machine Learning for Green Solvents: Assessment, Selection and Substitution

**DOI:** 10.1002/advs.202516851

**Published:** 2025-11-16

**Authors:** Rohan Datta, Janhavi Nistane, Abhishek Sose, Harikrishna Sahu, Rampi Ramprasad

**Affiliations:** ^1^ School of Chemical and Biomolecular Engineering Georgia Institute of Technology Atlanta Georgia 30332 USA; ^2^ School of Materials Science and Engineering Georgia Institute of Technology Atlanta Georgia 30332 USA

**Keywords:** green solvents, machine learning

## Abstract

Strict environmental regulations have intensified the demand for green solvents that can replace hazardous ones without compromising performance. Existing methods for estimating solvent sustainability rely on Solvent Selection Guides (SSGs), which assign scores based on environmental, health, safety, and waste (EHSW) criteria, covering as few as 200 solvents. Expanding these guides is tedious, as it requires over 30 properties per solvent, many of which are often unavailable. Moreover, identifying greener alternatives within the limited SSG pool is challenging due to the need to balance conflicting criteria such as sustainability, cost, and performance. To address these limitations, a data‐driven pipeline is presented for assessing the sustainability of solvents and identifying greener substitutes. Three models are trained and evaluated on the GlaxoSmithKline Solvent Sustainability Guide (GSK SSG) to predict “greenness” metrics: a traditional Gaussian Process Regression (GPR) model, a fine‐tuned GPT model (FT GPT), and a GPT model using in‐context learning (ICL). It is found that GPR slightly outperforms language‐based GPT models and is used to evaluate 10,189 solvents, forming GreenSolventDB–the largest public database of green solvent metrics. These predictions are combined with Hansen solubility parameter‐based metrics to identify greener solvents with solubility behavior similar to hazardous solvents. This approach is validated through case studies on benzene and diethyl ether, with predicted alternatives aligning well with known greener substitutes. Building on this success, novel alternatives are proposed for the hazardous solvents listed in the GSK SSG. This framework for quantifying solvent sustainability and identifying greener substitutes is expected to significantly accelerate the discovery and adoption of environmentally‐friendly solvents.

## Graphical abstract

Environmental regulations have intensified demand for green solvents, but discovery is limited by Solvent Selection Guides (SSGs) that quantify solvent sustainability. Training a machine learning model on GlaxoSmithKline SSG, a database of sustainability metrics for 10,189 solvents, GreenSolventDB is developed. Integrated with Hansen solubility metrics, the pipeline identifies greener solvents with solubility similar to hazardous ones, accelerating green solvent discovery.

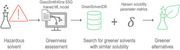

## 1 Introduction

With an annual global production of 20 million metric tons and a market value of USD 25 billion, organic solvents are essential to modern industry, supporting diverse applications in chemical manufacturing, pharmaceuticals, cosmetics, paints, and coatings.^[^ ^1^, ^2^ ^]^ However, many commonly used solvents pose significant toxicity and safety risks. Growing awareness of these hazards has led to increased regulatory scrutiny and impending bans, such as that of carcinogenic methylene chloride,^[^ ^3^ ^]^ under frameworks like the EU's REACH regulation^[^ ^4^ ^]^ and the U.S. EPA's green chemistry initiatives.^[^ ^5^ ^]^ Beyond health, safety and environmental concerns, the handling and disposal of hazardous solvents also carry substantial economic costs. As a result, tightening regulations, rising disposal expenses, and escalating sustainability demands are no longer just challenges–they are powerful drivers accelerating the transition to greener, more sustainable solvent substitutes.

Although the definition of “green” solvents remains somewhat ambiguous in the literature, there is general consensus that such solvents should minimize environmental, health, and safety hazards, while also reducing energy demands across their lifecycle–from manufacture to use and disposal.^[^ ^6^, ^7^, ^8^, ^9^, ^10^, ^11^, ^12^ ^]^ Historically, qualitative assessments of health, safety, and environmental impact have been compiled through frameworks such as the Globally Harmonized System of Classification and Labelling of Chemicals (GHS).^[^ ^13^ ^]^ Based on this database, along with additional considerations for industrial constraints, various organizations and pharmaceutical companies have developed Solvent Selection Guides (SSGs), which rank commonly used organic solvents using color‐coded systems (e.g., green for preferred, yellow for acceptable, red for undesirable) or numerical scoring (usually on a scale of 1–10) based on environmental, health, safety, waste (EHSW), life‐cycle assessment, and company‐specific criteria. Several widely adopted SSGs include those developed by Pfizer,^[^ ^14^ ^]^ AstraZeneca,^[^ ^15^ ^]^ Sanofi,^[^ ^16^ ^]^ GCI‐PR,^[^ ^17^, ^18^ ^]^ and GlaxoSmithKline.^[^ ^19^, ^20^, ^21^ ^]^ While these guides differ in their specific scoring criteria, prior studies have reported strong overall agreement among them.^[^ ^15^, ^22^ ^]^ Beyond SSGs, various specialized software tools have been developed to evaluate solvent sustainability,^[^ ^23^ ^]^ providing interactive platforms and rapid calculation of sustainability metrics. Some of these tools extend existing SSG frameworks,^[^ ^11^, ^17^, ^24^ ^]^ while others target specific applications such as analytical method selection, including softwares like GreenMotion,^[^ ^25^ ^]^ ComplexGAPI,^[^ ^26^ ^]^ and openLCA.^[^ ^27^ ^]^ However, these tools often rely on extensive input data that is not readily available, making it difficult to accurately assess the sustainability of new solvents and, in turn, to identify greener substitutes.

Historically, greener substitute solvents for hazardous substances were identified from a limited pool within SSGs and filtered to exhibit solubility similarities to the target hazardous solvent, using descriptors such as Hansen Solubility Parameters (HSP), Kamlet–Taft parameters, or solvatochromic properties.^[^ ^28^, ^29^ ^]^ For example, solvents were selected based on minimal differences in HSP, following the “like dissolves like” principle, where a smaller HSP difference indicates a closer solubility match, suggesting that one solvent can effectively replace another in applications where solubility is a key factor. In a recent study, Larsen et al. combined HSP‐based similarity with sustainability metrics from the GlaxoSmithKline Solvent Selection Guide (GSK SSG) to identify greener solvents for printed electronics, successfully validating two alternatives.^[^ ^11^ ^]^ However, their approach was constrained by a limited solvent pool (154 solvents), restricting exploration of broader chemical space and potentially overlooking other viable alternatives. While advancements in computational tools, such as COSMO‐RS (Conductor‐like Screening Model for Real Solvents), have provided more accurate estimates of molecular interactions and solvent properties (for example, HSP),^[^ ^30^, ^31^, ^32^ ^]^ the chemical space covered by SSGs for green solvent discovery remains relatively constricted. This limitation in constrained chemical space becomes particularly challenging when finding alternative green solvents for specific applications, as replacements must meet multiple–and often conflicting–criteria, such as sustainability, solubility, cost, and application‐specific performance,^[^ ^6^, ^16^ ^]^ highlighting the need to broaden the search beyond traditional SSGs. While expanding existing SSGs to include more solvents is one possible solution, this process is slow and labor‐intensive; for instance, the GSK SSG relies on over 30 solvent‐specific properties, making the addition of new solvents resource‐intensive.

Alternatively, machine learning (ML) algorithms present a promising alternative to traditional solvent selection strategies by enabling the prediction of solvent sustainability and the exploration of a much broader chemical space to identify greener alternatives. ML approaches have already demonstrated success in predicting solvent properties and behavior directly from molecular structure, significantly accelerating materials discovery and design.^[^ ^33^, ^34^, ^35^, ^36^ ^]^ While many ML‐based methods have been developed for green solvent identification, most rely on property‐rich datasets and clustering techniques, such as principal component analysis (PCA), to group chemically similar solvents within a limited chemical space, from which potentially more greener alternatives can be identified.^[^ ^17^, ^37^, ^38^, ^39^, ^40^, ^41^ ^]^ Notable open‐source tools for green solvent selection from AstraZeneca^[^ ^37^ ^]^ and the ACS Green Chemistry Institute^[^ ^17^ ^]^ use PCA‐based clustering to group solvents within a fixed pool of 292 candidates based on 30 properties. In contrast, the SUSSOL tool by Sels et al.^[^ ^38^ ^]^ represents another key advancement, allowing the clustering of new, unseen solvent datasets outside the traditional SSG domain, provided that the 22 physicochemical property data are available for these solvents. While these tools have significantly aided solvent substitution efforts, the limited chemical space and their reliance on extensive property data restricts their utility, particularly when experimental data are sparse or unavailable.

**Figure 1:**
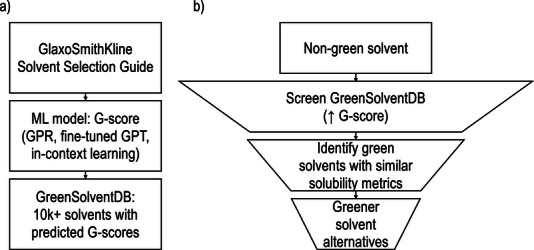
Workflow for predicting solvent sustainability and green solvent substitution: a) ML‐driven solvent sustainability prediction: A machine learning model is trained on the GSK SSG to predict solvent greenness, quantified by the G‐score. Next, we compile GreenSolventDB, a database of 10,189 solvents with ML‐predicted sustainability G‐scores. b) Green Solvent Substitution: For a given “non‐green” solvent, a greener alternative is identified from GreenSolventDB based on a higher predicted G‐score. Candidates are further filtered using HSP‐based metrics to ensure similar solubility behavior, enabling the identification of a pool of greener solvents suitable for drop‐in replacement.

To address the gap in greener solvent selection, we propose a data‐driven pipeline that i) quantitatively assesses the sustainability of novel solvents, even in the absence of complete physicochemical data, and ii) identifies greener substitutes to hazardous solvents (**Figure** **1**). Hypothesizing that solvent sustainability is linked to chemical structure, we trained a Quantitative Structure–Property Relationship (QSPR) based Gaussian Process Regression (GPR) ML model to map molecular fingerprints to sustainability metrics (namely, the composite sustainability score or G‐score) from the comprehensive GSK SSG. Alongside traditional QSPR models, we explored large language models (LLMs) like OpenAI's standard GPT‐3.5 Turbo for predicting solvent sustainability metrics, leveraging their success in molecular property prediction, ability to incorporate prior knowledge from literature, and bypass explicit molecular fingerprinting.^[^ ^42^, ^43^, ^44^, ^45^, ^46^, ^47^, ^48^, ^49^ ^]^ We evaluated GPT‐3.5 Turbo via fine‐tuning and in‐context learning, finding that although LLMs perform comparably, the GPR model still outperforms them in G‐score prediction. Building on this, we used the GPR model to predict sustainability metrics for over 10,189 solvents, creating GreenSolventDB, the largest publicly available database of green solvent metrics (Figure 1a). Additionally, we perform fragment‐level Z‐score analysis to identify structural motifs that influence predicted sustainability, aligning with chemical intuition and thus supporting our hypothesis that molecular structure influences sustainability. Next, we identify greener alternatives for a target solvent by querying GreenSolventDB for solvents with a higher G‐score. We then filter these solvents based on minimal differences in Hansen Solubility Parameters (HSP) based metrics, using the “like dissolves like” principle to find greener alternative solvents for existing applications with similar solubility behavior (Figure 1b). To validate our methodology, we conducted case studies on benzene and diethyl ether, two widely used hazardous solvents, for which our predicted greener alternatives align with the literature, indicating the reliability of our approach. We also propose undiscovered greener drop‐in replacements for 29 undesirable solvents listed in the GSK SSG, including benzene and diethyl ether, with these alternatives made available at our Github (https://github.com/Ramprasad‐Group/green_solvents/tree/main). This data‐driven pipeline, which quantifies solvent sustainability and identifies greener substitutes, along with the creation of GreenSolventDB, is expected to significantly accelerate the adoption of greener solvents by facilitating the discovery of greener alternatives.

## 2 Methods and Datasets

### 2.1 Training Dataset

**Figure 2:**
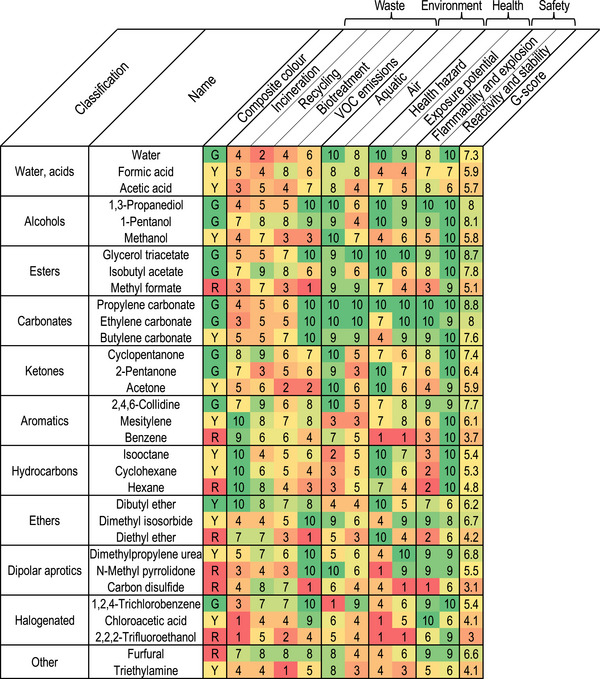
A snippet of the GSK SSG: Solvent sustainability is quantified by the G‐score, calculated as the geometric mean of four empirical EHSW (Environment, Health, Safety, and Waste) scores, each derived from ten underlying subcategories. A composite color, based on a traffic light system, visually summarizes the G‐score and incorporates GSK‐specific considerations and data gaps to offer an at‐a‐glance assessment of sustainability.

We train our models using the expanded GSK SSG,^[^ ^19^, ^20^, ^21^ ^]^ which evaluates 154 solvents using a transparent and systematic scoring framework across various EHSW aspects, as shown in **Figure** **2**. Further, this widely adopted guide integrates life cycle assessment principles and adjusts for data uncertainty by penalizing solvents with limited data.^[^ ^11^ ^]^ Solvent sustainability is quantified using the composite sustainability score, named as the G‐score, which ranges from 1 to 10, with higher values indicating greater sustainability. Section S1 (Supporting Information) highlights the distribution of G‐scores across different chemical classes, where carbonates and esters tend to score higher, while halogenated solvents score lower due to their higher toxicity. The G‐score evaluates the overall environmental, health, and safety impact of solvents and is calculated hierarchically from four intermediate category scores–Waste, Environment, Health, and Safety–each of which is based on relevant subcategory scores.

(1) $$\begin{array}{cc} & \text{Waste}=\\ & \sqrt[{4}]{\text{Incineration}\times \text{Recycling}\times \text{Biotreatment}\times \text{VOC emissions}} \end{array}$$

(2) $$\begin{array}{cc} \text{Environment} & =\sqrt{\text{Air}\times \text{Aquatic}} \end{array}$$

(3) $$\begin{array}{cc} \text{Health} & =\sqrt{\text{Health hazard}\times \text{Exposure potential}} \end{array}$$

(4) $$\begin{array}{cc} \text{Safety} & =\sqrt{\text{Flammability and explosion}\times \text{Reactivity and stability}} \end{array}$$

where *Incineration*, *Recycling*, *Biotreatment*, *VOC* (volatile organic compound) *emissions*, *Air*, *Aquatic*, *Health hazard*, *Exposure potential*, *Flammability and explosion*, and *Reactivity and stability* are subcategory scores (Figure: 2).

The G‐score, calculated as the geometric mean of the four category scores:

(5) $$\text{G-score}=\sqrt[{4}]{\text{Waste}\times \text{Environment}\times \text{Health}\times \text{Safety}}$$

While useful as a benchmark, we also note several limitations of the GSK SSG–based G‐score. First, the G‐score is a semi‐quantitative metric, so small differences may not reflect meaningful distinctions, and values can shift as new solvents and data are incorporated. As Byrne et al. highlight, the guide may also penalize “unknowns,” which can disadvantage novel or less studied solvents, including potentially greener options.^[^ ^22^ ^]^ Finally, it does not consider solvent origin (like bio‐based solvents) and prioritizes pharmaceutical contexts, so its application to other domains requires careful adaptation. Nonetheless, given the widespread adoption of the GSK SSG, the G‐score offers a consistent and recognized benchmark for solvent sustainability and is used as a target property to quantify solvent sustainability for ML model training.

### 2.2 G‐Score Predictive Workflows

Three types of predictive workflows were considered for the prediction of solvent sustainability, quantified by the GSK SSG‐based G‐score:

- **Gaussian Process Regression (GPR) Model**:
  Based on the hypothesis that chemical structure is correlated with solvent sustainability, selecting a fingerprinting scheme that effectively captures molecular features is critical. We therefore used Morgan fingerprints, a widely benchmarked method for representing small molecules,^[^ ^50^, ^51^, ^52^, ^53^ ^]^ as input features for the machine learning models predicting the solvent composite sustainability score (G‐score), encoding topological substructural information as a 1024‐bit binary vector generated by iterative hashing of circular atom neighborhoods up to radius 2.^[^ ^54^, ^55^ ^]^ While numerous alternative machine learning algorithms exist, we use GPR as it is particularly well‐suited for small datasets and, most importantly, because it provides uncertainty estimates, which are crucial when working with limited data, as they guide the cautious interpretation of any extrapolations beyond this regime. Hence, a Morgan‐fingerprint‐based five‐fold cross‐validated GPR model was developed using the *PolymRize* platform (https://polymrize.matmerize.com/), a standardized software for molecular and polymer informatics.
- **Fine‐tuned GPT‐3.5 Turbo Models (FT GPT)**: Fine‐tuning LLMs enables them to adapt to specific domains or tasks by updating a part of the model weights based on new training data. At the time this study was conducted, we employed GPT‐3.5‐Turbo, as it represented a practical middle ground between cost and comparable performance relative to GPT‐4‐based models (Section S2, Supporting Information) and it was fine‐tuned using the GSK SSG to specialize the model for solvent sustainability prediction. The GSK SSG dataset was reformatted into an instruction‐tuning prompt that was deliberately descriptive, since the G‐score is not a fundamental molecular property, as illustrated below:
User: As an expert in green chemistry, predict the GlaxoSmithKline (GSK) Solvent Sustainability Guide‐based Composite Scores. The GSK‐based Composite Scores are derived from environmental, health, waste, safety, and life cycle assessment considerations. A higher GSK Composite Score indicates a greener solvent, and a lower score indicates a less sustainable solvent. Predict the GSK Composite Score [Range: 1–10] for <SMILES string>
Assistant: <G‐score>
  Given the importance of prompt engineering, additional prompt variations are described in the Section S2 (Supporting Information). We note that the black‐box nature of fine‐tuning OpenAI's GPT models offers limited customization for hyperparameters, and the exact mechanism of fine‐tuning remains undisclosed.^[^ ^56^ ^]^ Fine‐tuning was restricted to modifying the number of training epochs (10, to prevent overfitting).
- **In‐Context Learning with GPT‐3.5 Turbo (ICL GPT)**:
  In‐context learning (ICL) typically refers to few‐shot or one‐shot prompting. Unlike fine‐tuning, ICL leverages pre‐trained models and operates without modifying model parameters and weights. The model learns from the context provided in the prompt, allowing the model to generate predictions based on retrieved information and on‐the‐fly learning. Here, “shots” indicate the number of examples shown to the model. The prompt format was kept consistent with the fine‐tuning setup, but with the inclusion of a few‐shot learning examples within the prompt itself (an example provided in the Section S2, Supporting Information)

Regarding the inference settings for the GPT models, we used a temperature (*T* = 0.5) to balance deterministic and probabilistic behavior in the predictions, while all other parameters were kept at their default values. Model performance was evaluated using Root Mean Square Error (RMSE) and Pearson correlation coefficient (r).

### 2.3 Compatible Solvent Selection

Hansen Solubility Parameters (HSP) are used to evaluate solvent compatibility based on cohesive energy densities, calculated in this work using the HSPiP software (Hansen Solubility Parameters in Practice).^[^ ^57^, ^58^ ^]^ The total or Hildebrand solubility parameter (δ) is described by Hansen using three components: δ_*D*_ (dispersion), δ_*P*_ (polar), and δ_*H*_ (hydrogen bonding):^[^ ^57^ ^]^

(6) $$\delta =\sqrt{\delta_{D}^{2}+\delta_{P}^{2}+\delta_{H}^{2}}$$

Solvents can be represented as points in a 3D Hansen space with axes 2δ_*D*_, δ_*P*_, and δ_*H*_. The Hansen solubility distance *R*_*a*_ between two solvents (denoted by subscripts 1 and 2) quantifies their solubility similarity:

(7) $$R_{a}=\sqrt{4{\left(\delta_{D1}-\delta_{D2}\right)}^{2}+{\left(\delta_{P1}-\delta_{P2}\right)}^{2}+{\left(\delta_{H1}-\delta_{H2}\right)}^{2}}$$

While a smaller *R*_*a*_ suggests similar solubility behavior, the Relative Energy Difference (RED) must also be taken into account.^[^ ^59^ ^]^ RED is *R*_*a*_ normalized by the interaction radius *R*_0_, which defines the solubility boundary for a given solute.

(8) $$\text{RED}=\frac{R_{a}}{R_{0}}$$

*R*_0_ is typically obtained by fitting a solubility sphere that includes all “good” solvents and excludes “poor” ones based on experimental visual observation of the solvents (usually 20–30).^[^ ^60^, ^61^ ^]^ Hence, *R*_0_ depends on both the solute and the number of solvents tested. When experimental fitting is not feasible, a fixed value is often used. In this study, we adopt a conservative default value of *R*_0_ = 4, thereby restricting compatibility to solvents that are highly similar to the target.^[^ ^62^ ^]^ Hence, by using RED as the HSP‐based metric to quantify solubility similarity, we identify safer drop‐in alternatives to hazardous solvents by selecting candidates with RED < 1, where smaller RED values indicate stronger solubility‐based compatibility.

### 2.4 Z‐Score Analysis

To gain insight into how the production‐level ML model predicts G‐scores based on molecular fragments, we performed a Z‐score analysis using the hypergeometric distribution. The molecular fragments were derived using BRICS (breaking of retrosynthetically interesting chemical substructures)^[^ ^63^ ^]^ decomposition of solvent molecules. The hypergeometric distribution estimates the probability of observing *k* instances of a fragment in a subset of *n* molecules, given that the fragment appears *K* times in the full dataset of size *N*.^[^ ^64^, ^65^, ^66^ ^]^ The enrichment or depletion of each fragment was quantified using a Z‐score (*Z*), calculated as:

(9) $$Z=\frac{k-\langle k\rangle }{\sigma (k)}$$

where $\left\langle k\right\rangle =\frac{nK}{N}$ is the expected count, and σ(*k*) is the standard deviation of the hypergeometric distribution. A positive Z‐score indicates that a fragment is enriched (appears more frequently than expected by chance), while a negative Z‐score suggests underrepresentation.

## 3 Results

### 3.1 ML Model Performance

**Figure 3:**
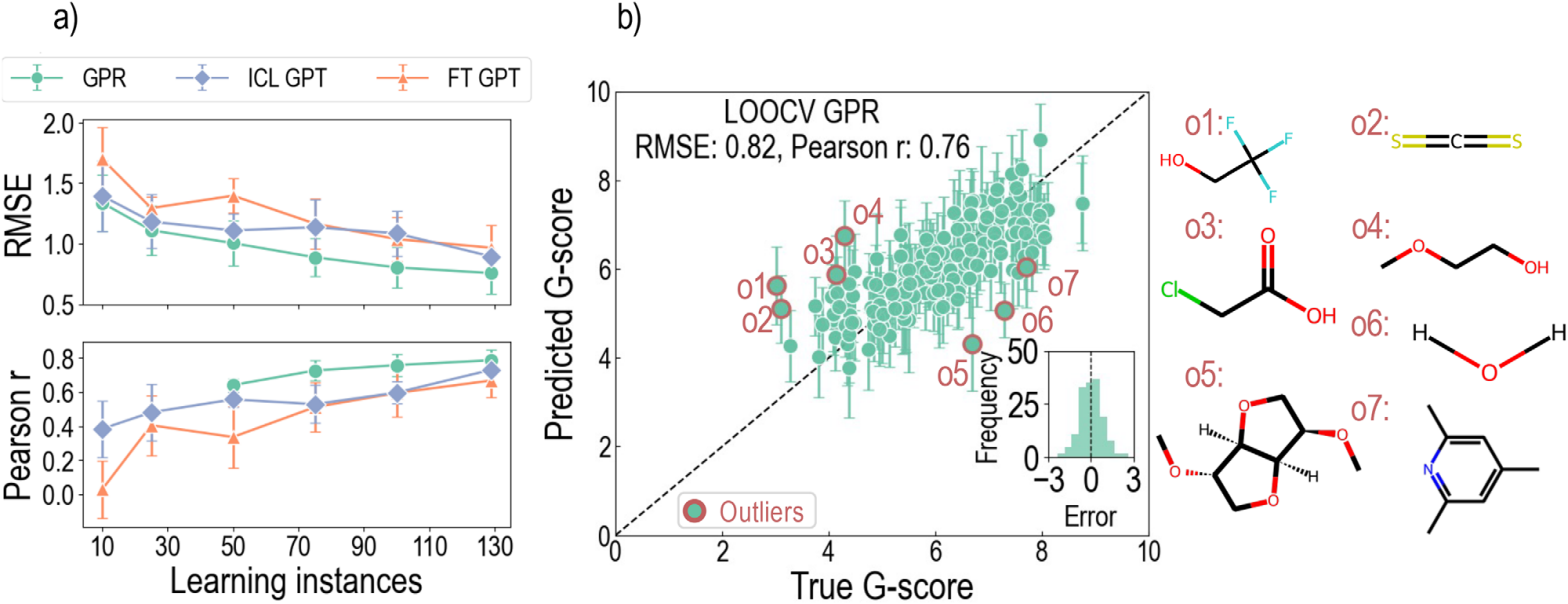
Model performance: a) Learning curves showing test set error metrics versus number of learning instances (which is the training size for GPR and fine‐tuned GPT models, and number of shots for in‐context learning (ICL)). Error metrics are averaged over five runs with nested subsets and a fixed test set. Here, the GPR model shows the lowest error among these models at higher training sizes and serves as the basis for the final production model. b) LOOCV parity plot for the best‐in‐class GPR model with error bars indicating prediction uncertainty. The inset shows a near‐normal distribution of prediction errors (difference between the ground truth and predicted values). The seven identified outliers (o1–o7) are highlighted in red, with their chemical structures shown on the right.

We evaluated the performance of the three predictive models–Gaussian Process Regression (GPR), fine‐tuned GPT (FT GPT), and in‐context learning GPT (ICL GPT)–for predicting sustainability metrics (G‐scores). To assess model accuracy and overfitting, we analyzed learning curves (**Figure** **3**), plotting the average test RMSE and Pearson r as a function of the number of learning instances. The number of learning instances was defined as the number of training examples for the GPR and FT GPT models, and as the number of shots for the ICL GPT model. The dataset was shuffled and randomly split to create fixed unseen test sets of 25 datapoints. Models were incrementally evaluated with increasing numbers of learning instances (10, 30, 50, 75, etc.), with each larger set containing all previous instances, and predictive performance was recorded on the same unseen test set. For each learning instance size, five independent random splits were performed, and the reported error metrics represent the average across these runs. As shown in Figure 3a, all three models demonstrated improved predictive performance with increasing training size, as evidenced by decreasing RMSE and increasing Pearson r values. Notably, for the GPR model, Pearson correlation could not be defined for fewer than 50 training instances, as the model tended to predict a constant value under extremely data‐scarce conditions. In contrast, the ICL GPT model performed best in such low‐data regimes, followed by the FT GPT model, likely benefiting from prior knowledge encoded in the large language model. As the number of training instances increased, all three models showed converging performance, with GPR ultimately marginally outperforming the language models. These analyses support our hypothesis that G‐scores follow a strong QSPR relationship, making GPR a suitable choice for our production‐level models.

Additionally, in Section S3 (Supporting Information), we contrast the performance of GPR and large language models in predicting EHSW scores based on the GSK SSG. All predictive approaches demonstrate comparable performance, with GPR exhibiting marginally better predictive performance. Although the G‐score can be derived as the geometric mean of the EHSW scores, this method was not adopted due to its complexity and comparable performance relative to models trained directly on the G‐score (Section S2, Supporting Information).

To further evaluate the GPR model's ability to predict G‐scores for new solvents–its intended application–we performed leave‐one‐out cross‐validation (LOOCV). This approach is well‐suited for small datasets, as it maximizes training data usage while testing generalizability. As shown in Figure 3b, the LOOCV GPR model achieved an RMSE of 0.82 (or a Mean Absolute Error of 0.65) indicates that predictions are within 15% values, while the slightly lower value of the coefficient of determination R^2^ (0.58) was observed due to the sensitivity of this metric to outliers. Instead, the more appropriate metric is the Pearson correlation (0.76), which is more robust to outliers, indicating a satisfactory agreement between experimental and predicted values, as can also be seen in the LOOCV parity plot (Figure 3b), with a fairly tight distribution. To further assess model limitations, we analyzed outliers in the LOOCV GPR solvent predictions by calculating the error between predicted and target G‐scores and identifying points more than two standard deviations from the mean. Seven outliers (o1–o7) were found, each with a unique chemical profile likely explaining the model's difficulty in generalizing; detailed explanations are provided in Section S4 (Supporting Information).

### 3.2 GreenSolventDB: Expansion in Chemical Space

**Figure 4:**
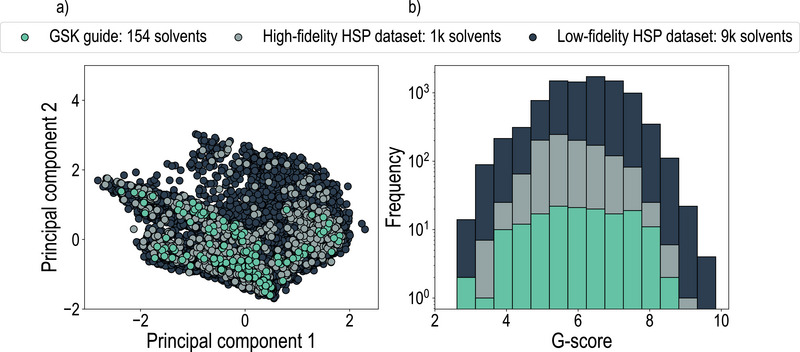
Expansion of chemical space for green solvent substitution: a) Principal Component Analysis (PCA) illustrates the expansion of chemical space from the limited set of 154 solvents in the GSK SSG to the more diverse GreenSolventDB. GreenSolventDB includes 1155 solvents with high‐fidelity experimental HSPs and an extended superset of 9034 solvents with lower‐fidelity, estimated HSPs. b) G‐score distribution: true G‐score distribution for the GSK SSG and the predicted G‐score distribution across the broader GreenSolventDB.

We developed GreenSolventDB, a comprehensive database of 10,189 solvents–the largest to date with predicted sustainability metrics (G‐scores) generated using a production‐level GPR model. GreenSolventDB was built using a dataset from the HSPiP software,^[^ ^58^ ^]^ comprising 1,155 high‐fidelity solvents with experimentally determined or validated HSPs and 9,034 lower‐fidelity solvents with HSPs estimated via the Yamamoto Molecular Break (Y‐MB) group contribution method, which decomposes molecular structures into fragments to predict dispersion, polar, and hydrogen‐bonding components.^[^ ^67^, ^68^ ^]^ We acknowledge potential limitations in the accuracy of lower‐fidelity set, as the Y‐MB method is less reliable for large molecules. Due to licensing restrictions, the HSP values are not included in GreenSolventDB; however, we provide the predicted G‐scores for all 10,189 solvents (Data Availability Statement Section). This resource significantly expands the accessible chemical space for green solvent discovery, going far beyond traditional solvent selection guides such as the GSK SSG, which includes fewer than 200 solvents. To visualize the expansion of chemical space, we performed principal component analysis (PCA) on Morgan fingerprints generated from SMILES strings using a radius of two bonds and a 1024‐bit vector representation.^[^ ^50^, ^51^, ^52^, ^53^ ^]^ The fingerprints were reduced to two principal components using the scikit‐learn library^[^ ^69^ ^]^ (version 1.6.1), capturing the largest variance and providing a structured visualization of chemical space, where each point represents a unique solvent (**Figure** **4**a). Additionally, the true G‐score distribution observed in the GSK SSG is closely mirrored by the predicted G‐scores across GreenSolventDB (Figure 4b), with some solvents extending toward the higher G‐score range, hinting that numerous undiscovered greener solvent candidates may exist within this expanded landscape.

### 3.3 Z‐Score Analysis: ML Model Interpretation

**Figure 5:**
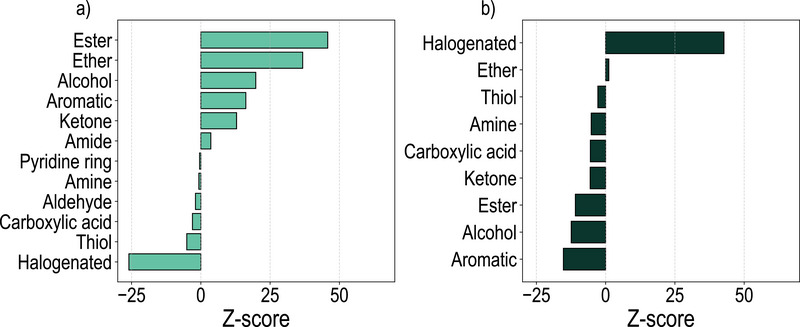
Z‐score analysis of ML‐predicted sustainability. Fragment enrichment in: a) green solvents (G‐score > 7), dominated by esters and alcohols, and b) non‐green solvents (G‐score < 4.5), enriched in toxic halogenated groups.

To understand how the production level GPR ML model assigns G‐scores based on molecular fragments, we performed a hypergeometric Z‐score (Z) analysis comparing fragment enrichment in green (say, G‐score > 7) versus non‐green (G‐score < 4.5) solvents in GreenSolventDB (**Figure** **5**). Consistent with Section S1 (Supporting Information), green solvents showed strong enrichment for specific fragments such as esters (Z = 45.77) and alcohols (Z = 19.81), in line with known trends from the literature.^[^ ^22^ ^]^ On the other hand, as expected, fragments associated with halogens were largely underrepresented (Z = ‐25.97) in green solvents, reflecting their known toxicity.^[^ ^70^, ^71^ ^]^ Unexpectedly, ethers (Z = 36.74) also showed a positive Z‐score, which may be due to their frequent co‐occurrence with other functional groups that more strongly influence the model's sustainability predictions.

Conversely, these halogenated fragments (Z = 42.70) and ethers (Z = 1.165) were enriched in non‐green solvents (predicted G‐scores < 4.5). Furthermore, fragments such as alcohols (Z = ‐12.40), aromatics (Z = ‐15.26), and esters (Z = ‐10.94) were significantly underrepresented in solvents with low G‐scores, reinforcing their strong association with higher ML‐predicted sustainability. Additionally, sulfur‐ and nitrogen‐containing fragments showed negative Z‐scores in both sustainable and unsustainable solvent groups, suggesting that these fragments are more common in solvents with intermediate G‐scores. Overall, the Z‐score analysis is consistent with chemical intuition and literature findings regarding which molecular fragments may contribute to solvent sustainability, thereby reinforcing the credibility and interpretability of the ML model.

### 3.4 Green Solvent Substitution and Discovery

#### 3.4.1 Greener Alternatives for Hazardous Solvents in the GSK SSG

Using the pipeline shown in Figure 1, we identified greener alternatives (with RED values <1, ideally near 0, and higher predicted G‐scores) for 29 hazardous solvents from the GSK SSG by screening over 10,189 candidates in GreenSolventDB. Several literature‐supported substitutions were re‐discovered, with key results summarized in **Table** **1**.

**Table 1:** Selected greener alternatives for non‐green solvents, identified by our pipeline and validated by literature. For each solvent, key hazards are summarized, along with both literature‐supported and predicted substitutes. The number of potentially greener candidates identified from GreenSolventDB are also reported.

Non‐green solvent	Issues	# Green alternatives	Selected greener alternatives also found in the literature
Pentane	Toxicity (reproductive hazards), low flash point (flammability)	592	Heptane^[^ ^14^, ^72^, ^73^, ^74^ ^]^
Tetrahydrofuran/THF	Peroxide formation, flammability	1952	Cyclopentyl methyl ether/CPME^[^ ^72^, ^73^, ^74^, ^75^ ^]^
N‐methyl pyrrolidone/NMP	Toxicity	317	N‐butyl pyrrolidone^[^ ^14^, ^73^ ^]^
Benzene	Hazardous airborne pollutant (HAP), Toxicity (carcinogen)	629	Toluene^[^ ^14^, ^76^, ^77^, ^78^ ^]^
Diethyl ether	Low flash point, peroxide formation, high volatility	1520	Methyl tert‐butyl ether/MTBE, Diethylene glycol dibutyl ether/DGDE^[^ ^79^, ^80^ ^]^
1,2‐Dichloroethane	HAP, Toxicity (carcinogen)	1882	Dichloromethane^[^ ^72^ ^]^
*Greener alternatives for other GSK SSG hazardous solvents, including those above, are detailed in the Data Availability Statement section*.			

For example, heptane (RED: 0.4, predicted G‐score: 5.42) is a widely recognized greener alternative to pentane (predicted G‐score: 5.11), supported by both literature and our pipeline.^[^ ^14^, ^72^, ^73^, ^74^ ^]^ Beyond pentane's well‐known toxicity, its extremely low flash point (–49°C) presents serious flammability risks, reflected in its low GSK flammability and explosivity scores.^[^ ^14^, ^19^ ^]^ While not entirely benign, heptane offers a safer profile with a higher flash point (–4°C) and reduced toxicity, making it a preferred substitute.^[^ ^14^, ^72^, ^73^, ^74^ ^]^ Similarly, tetrahydrofuran/THF (G‐score: 4.98) is a carcinogen and peroxide former; consistent with literature, we identified cyclopentyl methyl ether/CPME (RED: 0.99, G‐score: 5.12) as a safer alternative with lower genotoxicity and reduced peroxide formation.^[^ ^72^, ^73^, ^74^, ^75^ ^]^ We also highlight n‐butyl pyrrolidone (RED: 0.74, G‐score: 5.92) as a less toxic replacement for n‐methylpyrrolidone/NMP (G‐score: 5.642).

Apart from the validation cases in Table 1, we critically examine false positives and false negatives in green solvent identification. For instance, acetonitrile (G‐score: 5.656) is often cited as a greener alternative to NMP (G‐score: 5.642), but our pipeline excluded it due to its high RED value (2.06). Similarly, dichloromethane (G‐score: 4.776) in extraction is sometimes replaced with ethyl acetate (RED: 0.78, G‐score: 6.975), methyl tert‐butyl ether/MTBE (RED: 1.431, G‐score: 5.173), toluene (RED: 2.013, G‐score: 5.96), or 2‐methyltetrahydrofuran/2‐MeTHF (RED: 0.907, G‐score: 4.71).^[^ ^72^, ^73^, ^74^, ^75^ ^]^ While ethyl acetate is correctly identified, the other alternatives MTBE and toluene exceed our RED cutoff and are excluded, introducing potential false negatives. This highlights the distinction of our RED‐based criterion, which deliberately prioritizes solubility similarity, in contrast to literature guides that largely emphasize functional‐similarity‐based substitutions. Additional false negatives arise when the semi‐quantitative G‐score differences are small‐for example, our pipeline incorrectly favors hexane (G‐score: 5.474) over heptane (RED: 0.2, G‐score: 5.42), despite heptane being greener.^[^ ^72^, ^73^, ^74^, ^75^ ^]^ Another case is 2‐MeTHF, widely labeled as greener alternative to some ethers in the literature due to its renewable biomass‐based origin, but classified as undesirable in the GSK guide owing to toxicity and flammability, which justifies its exclusion.^[^ ^22^, ^72^, ^73^, ^74^, ^75^ ^]^ False positives (pipeline‐suggested solvents that are not truly greener) are harder to detect. Candidates must be evaluated for downstream use and hazards; for example, cyclohexylbenzene (RED: 0.29, G‐score: 6.30) was suggested as a benzene replacement but raises concerns due to reported aquatic toxicity.^[^ ^81^ ^]^ Such false positives may be frequent, as many candidates from GreenSolventDB are suggested based on only incrementally higher G‐scores. This highlights the need to downselect solvents not just by requiring substantial increments in G‐score, but also through careful evaluation of their suitability for downstream applications.

Despite these limitations, validation across numerous cases in Table 1, along with Section S5 (Supporting Information) (Table 1), which presents high G‐score predictions for recently reported green solvents, as expected, provides additional evidence of the pipeline's ability to identify greener alternative solvents. We further examine two case studies–greener substitutes for benzene and diethyl ether, to examine the strengths and limitations of our data‐driven pipeline.

#### 3.4.2 Case Study 1: Greener Benzene Substitutes

Benzene is widely used as a solvent in chemical processes due to its low boiling point and excellent solvency for organic compounds, with applications ranging from organic synthesis to analytical chemistry.^[^ ^82^, ^83^ ^]^ However, benzene is a well‐established human carcinogen, with epidemiological studies linking exposure to hematological disorders such as leukemia.^[^ ^84^, ^85^, ^86^ ^]^ Due to the significant health risks associated with benzene exposure, identifying effective greener alternatives is imperative.^[^ ^87^ ^]^

**Figure 6:**
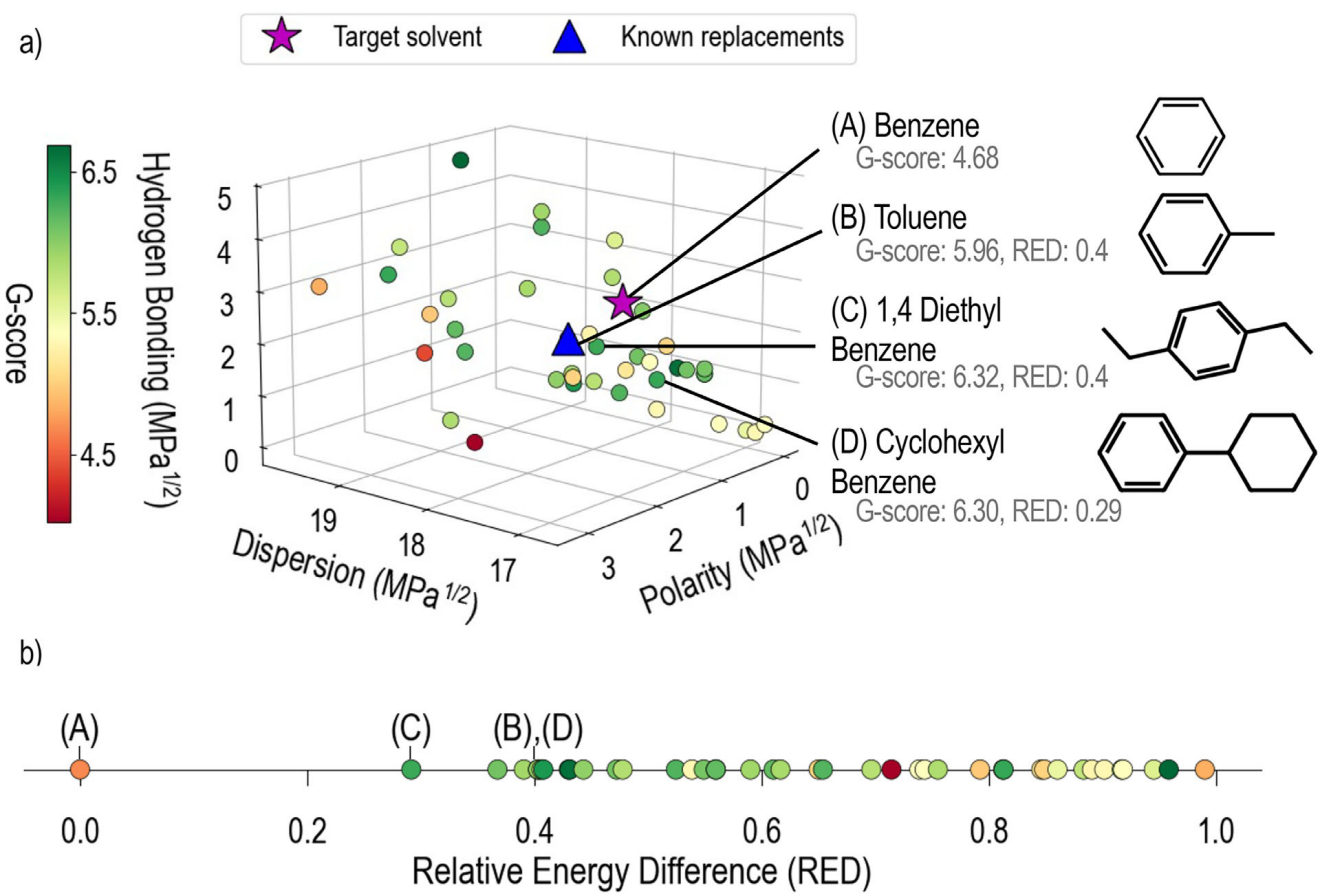
Greener benzene substitutes: a) 3D plot of solvents in Hansen space, showing the target solvent benzene (A, pink star) alongside alternative candidates from the high‐fidelity dataset with higher predicted G‐scores and RED < 1, color‐coded by their composite sustainability scores (G‐scores). b) Number line plot depicting the Relative Energy Difference (RED) between benzene and alternative candidates. Toluene (B), a well‐known alternative,^[^ ^14^, ^76^, ^77^, ^78^ ^]^ is identified by our ML pipeline, while 1,4‐DEB (C) and cyclohexyl benzene (D) are novel substitutes discovered through ML and not previously reported.

To find benzene alternatives (solvent A, **Figure** **6**) within the high‐fidelity GreenSolventDB search space, we screened for solvents with higher G‐scores and RED < 1 with respect to benzene, identifying candidates that are both greener and solubility‐compatible. Within the GreenSolventDB search space, toluene (solvent B, Figure 6) was identified as a potential replacement for benzene, with a predicted G‐score of 5.96 compared to benzene's 4.68, with a RED of 0.4, indicating its similarity to benzene. Toluene and benzene possess similar aromatic structures, and the addition of a methyl group in toluene, resulting in comparable solvating behavior. However, unlike benzene which upon entering the bloodstream, forms harmful metabolites linked to carcinogenicity and DNA damage, toluene produces less toxic, excretable compounds, making it a safer, non‐carcinogenic alternative.^[^ ^81^, ^88^ ^]^ As a result, toluene has been widely adopted as a safer solvent replacement for benzene.^[^ ^14^, ^76^, ^77^, ^78^ ^]^ The identification of toluene as an alternative to benzene through our pipeline and its alignment with established literature validate the effectiveness of our data‐driven approach. Beyond literature validation, our pipeline identifies unreported greener alternatives such as 1,4‐diethylbenzene/1,4‐DEB (solvent C, RED: 0.4, G‐score: 6.32), a low‐toxicity molecule.^[^ ^89^ ^]^ We also highlight cyclohexylbenzene (solvent D, RED: 0.29, G‐score: 6.30), which may require further scrutiny due to reported aquatic toxicity,^[^ ^81^ ^]^ underscoring the need for comprehensive evaluation prior to experimental validation (Likely a false positive, as pointed out in Section 3.4.1). In total, 629 potentially greener alternative candidates for benzene are provided in the Data Availability Statement section, of which 85 possess G‐scores greater than seven. These candidates require further evaluation for their intended downstream use to avoid false positives.

#### 3.4.3 Case Study 2: Greener Diethyl Ether Substitutes

**Figure 7:**
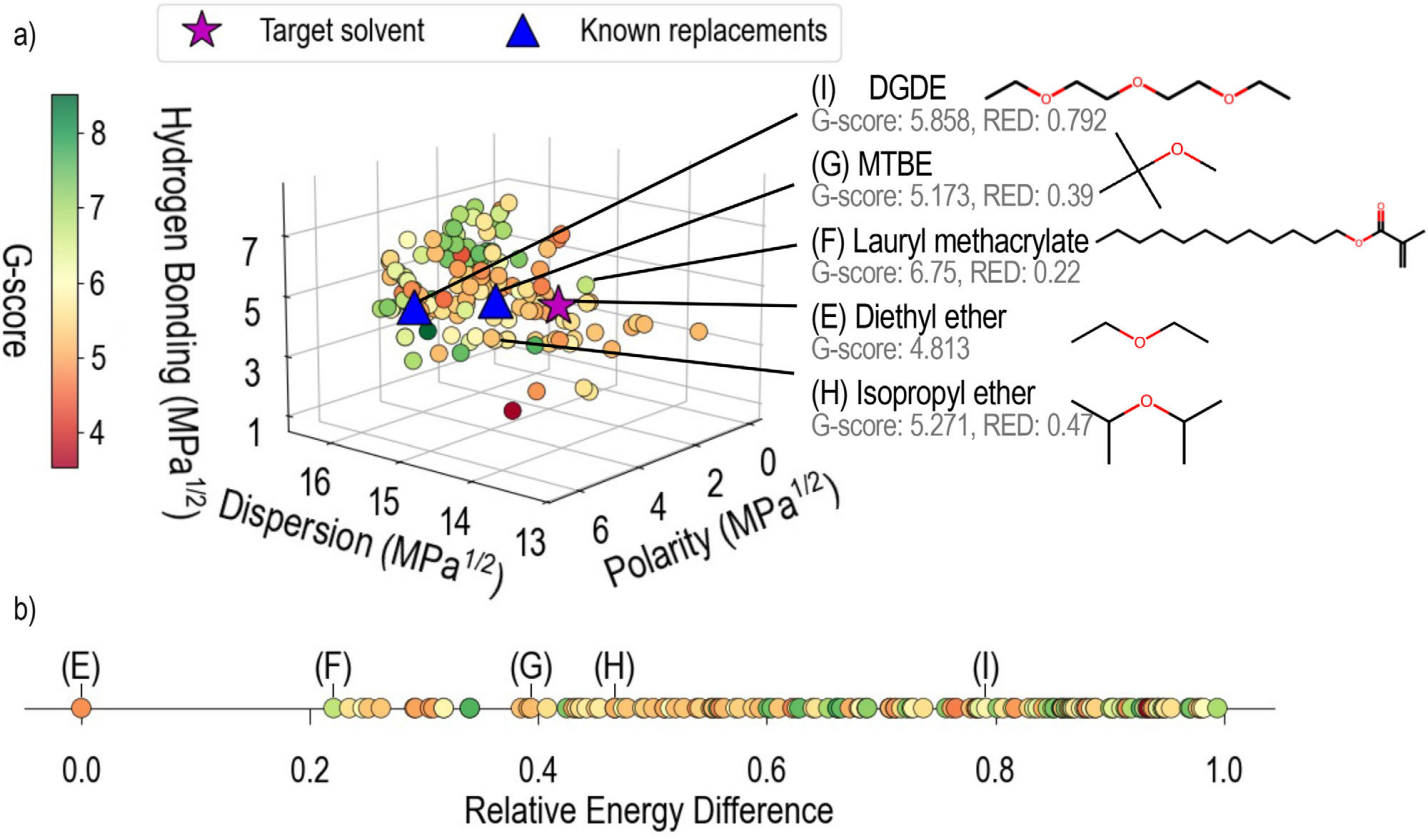
Greener diethyl ether substitutes: a) 3D plot of solvents in Hansen space, showing the target solvent diethyl ether (E, pink star) alongside alternative candidates from the high‐fidelity dataset with higher predicted G‐scores and RED < 1, color‐coded by their composite sustainability scores (G‐scores). b) Number line plot depicting the Relative Energy Difference (RED) between diethyl ether (E) and selected candidates. Methyl‐t‐butyl ether/MTBE (G), isopropyl ether (H), and diethylene glycol dibutyl ether/DGDE (I) are known alternatives^[^ ^14^, ^79^, ^80^ ^]^ identified by our ML pipeline, while lauryl methacrylate (F) is a novel ML‐discovered substitute not previously reported in the literature.

We next examine diethyl ether (solvent E, **Figure** **7**), a staple solvent in organic synthesis and extractions, as well as a general‐purpose laboratory solvent due to its excellent solvating abilities.^[^ ^90^ ^]^ However, it poses significant environmental, health, and safety (EHS) concerns: it is highly flammable with a flash point of ‐40°C, forms explosive peroxides upon air and light exposure during storage, and its low boiling point (34°C) causes high volatility, increasing inhalation risks that lead to dizziness, nausea, and respiratory irritation.^[^ ^14^, ^76^, ^77^, ^91^ ^]^

From the high‐fidelity GreenSolventDB search space, our computational pipeline identified methyl tert‐butyl ether/MTBE (solvent G, RED: 0.39, G‐score: 5.173) and diethylene glycol dibutyl ether/DGDE (solvent I, RED: 0.792, G‐score: 5.858) as viable replacements for diethyl ether (G‐score: 4.813). MTBE and DGDE are well‐documented in the literature as greener alternatives to diethyl ether due to their significantly lower volatility, higher flash points, reduced peroxide formation risk, and strong solvating capabilities.^[^ ^79^, ^80^ ^]^ Further, isopropyl ether (solvent H, RED: 0.47, G‐score: 5.271) is also recognized as a green solvent, although not as explicitly as diethyl ether replacement in sustainable chemistry guidelines.^[^ ^14^ ^]^ Additionally, we note a false negative: 2‐MeTHF (RED: 1.312, G‐score: 4.71), often cited as a greener diethyl ether alternative due to its biomass‐based origin, but classified as undesirable in the GSK pipeline owing to its toxicity and flammability.^[^ ^22^, ^72^, ^73^, ^74^, ^75^ ^]^

We also highlight unexplored potential alternatives, such as lauryl methacrylate (Solvent F; RED: 0.22, G‐score: 6.75). Its low volatility, attributed to a long alkyl chain, lowers flammability and VOC emissions,^[^ ^92^ ^]^ making it a promising substitute in niche applications such as UV‐curable coatings.^[^ ^93^ ^]^ However, due to this low volatility, lauryl methacrylate may be unsuitable for processes requiring rapid solvent removal–one of diethyl ether's primary advantages. This illustrates an important point: while the ML pipeline can suggest potential alternatives, detailed investigation of each candidate's properties is essential to assess suitability for specific applications. From the larger but lower‐fidelity GreenSolventDB space, tert‐butyl acetate/TBA (RED: 0.47, G‐score: 6.183) emerges as another attractive candidate, noted for its lower toxicity and reduced risk of peroxide formation–a significant safety concern associated with diethyl ether.^[^ ^94^ ^]^ In total, 1520 potentially greener alternatives for diethyl ether are linked in the Data availability section, with 364 candidates having predicted G‐scores greater than seven.

As detailed in these case studies, the alignment between our ML‐derived solvent recommendations and established green solvent literature validates the robustness and effectiveness of our computational screening pipeline. Additionally, we provide comprehensive candidate sets of greener alternatives for the hazardous solvents listed in the GSK SSG. While some alternatives may differ chemically or functionally and may not serve as exact drop‐in replacements, they offer valuable starting points for further screening and application‐specific optimization–especially for hazardous solvents that currently lack well‐established green substitutes.

## 4 Conclusion

In this work, we introduced a data‐driven pipeline to quantitatively assess solvent sustainability and identify greener “drop‐in” alternatives for undesirable solvents. We evaluated a range of predictive models trained on the GlaxoSmithKline Solvent Selection Guide (GSK SSG) to predict sustainability scores. We evaluated both traditional machine learning methods, including a Gaussian Process Regressor (GPR), and large language model (LLM)‐based approaches, such as a fine‐tuned GPT‐3.5 Turbo and in‐context learning. Among these, GPR consistently marginally outperformed the LLM‐based models and was ultimately selected as the final predictive model. Building on this, we created GreenSolventDB–a comprehensive database containing sustainability metrics for over 10,189 solvents, significantly expanding the chemical space beyond traditional SSGs. Additionally, to enhance model interpretability, fragment‐level Z‐score analysis was performed, revealing structural features associated with higher or lower sustainability and providing insight into the model's predictive behavior. Next, we identified greener drop‐in substitutes for several target non‐green solvents by querying GreenSolventDB and applying Hansen solubility parameter‐based metrics to ensure solubility behavior similar to that of the target solvents. The greener solvent candidates identified by our pipeline for benzene, diethyl ether, pentane, etc., closely match those reported in the literature, validating the robustness and reliability of our approach. Extending this work, we propose greener replacements for 29 undesirable solvents listed in the GSK SSG, many of which, to our knowledge, have not been previously reported. We emphasize that while these candidates exhibit strong sustainability potential, further evaluation of their performance and compatibility with specific applications is necessary before practical implementation. This work introduces a novel, data‐driven pipeline that quantifies solvent sustainability without elaborate physicochemical data, enabling rapid and scalable discovery of greener alternatives.^[^ ^95^, ^96^, ^97^ ^]^

## Conflict of Interest

R.R. is a founder of Matmerize, Inc., a company specializing in materials informatics software and services. Georgia Tech retains the rights to use PolymRize, the software produced by Matmerize, Inc., for research use. The other authors have no conflict of interest to declare.

## Author Contributions

R.D. and J.N. contributed equally to this work. The project was conceived and directed by R.R. R.D., and J.N contributed equally and conducted the data analysis, trained, and evaluated the machine learning models, and wrote the manuscript. A.S. trained some of the language models. H.S. collected the data and provided feedback and guidance. R.D., J.N., H.S., and R.R. discussed the results and reviewed the manuscript.

## Supporting information

Supporting Information

## Data Availability

The dataset from the GSK SSG, which was used to train the production‐level machine learning model, is available at https://github.com/Ramprasad‐Group/green_solvents/tree/main. In addition, this repository includes GreenSolventDB–predictions of G‐scores (sustainability metrics) for over 10,189 solvents. Due to licensing restrictions, HSP values are not included in the public version of GreenSolventDB. Additionally, we include green substitution candidate sets for the hazardous solvents identified in the GSK SSG.
