# Supplementary material for: Machine Learning for Green Solvents: Assessment, Selection and Substitution
Source: Adv Sci (Weinh). 2025 Nov 16:e16851. Online ahead of print. doi: 10.1002/advs.202516851 (PMC13325502; doi:10.1002/advs.202516851)
Supplement: Supplementary file 1 — Supporting Information [file ADVS-9999-e16851-s001.pdf]

Machine Learning for Green Solvents: Assessment, Selection and Substitution

Supplementary Information

Rohan Datta,[†,¶] Janhavi Nistane,[‡,¶] Abhishek Sose,[‡] Harikrishna Sahu,[‡] and Rampi Ramprasad[*,‡]

†*School of Chemical and Biomolecular Engineering, Georgia Institute of Technology, Atlanta, Georgia 30332, United States*

‡*School of Materials Science and Engineering, Georgia Institute of Technology, Atlanta, Georgia 30332, United States*

¶*These authors contributed equally to this work.*

E-mail: rampi.ramprasad@mse.gatech.edu

1 Training dataset

The distribution of solvent G-score across major chemical classes can be seen in Figure 1, highlighting trends in relative sustainability. Carbonates and esters generally exhibit higher G-scores, reflecting their favorable environmental and safety profiles[1], whereas halogenated solvents, which can be attributed to their toxicity.[2,3] These trends underscore the molecular structural influence on solvent sustainability.

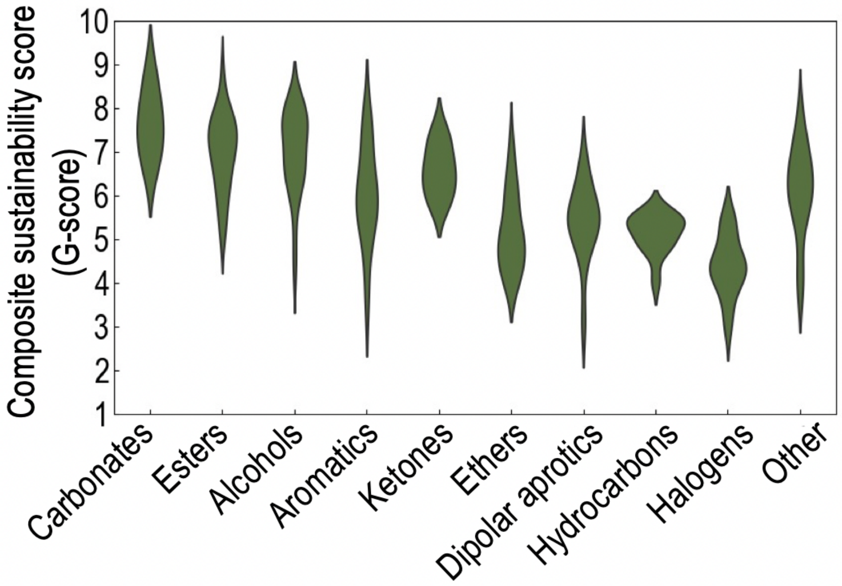

Figure 1: Distribution of 154 solvents in the expanded GSK Green Solvent Selection Guide, categorized by chemical class and composite sustainability score (G-score).

2 Prompt engineering and LLM model selection

Given the importance of prompt engineering for the performance of large language models, we highlight prompt used in this study with a 5-shot example. In addition, we explored several prompt variations, which are discussed below.

```
User: As an expert in green chemistry, predict the
GlaxoSmithKline (GSK) Solvent Sustainability Guide-based
Composite Scores. The GSK-based Composite Scores are derived from
environmental, health, waste, safety, and life cycle assessment
considerations. A higher GSK Composite Score indicates a greener
solvent, and a lower score indicates a less sustainable solvent.
Predict the GSK Composite Score [Range: 1-10] for CCCCC.
Assistant: 5.106
Predict the GSK Composite Score [Range: 1-10] for CCOCC.
Assistant: 4.813
Predict the GSK Composite Score [Range: 1-10] for OCCCO.
Assistant: 8.017
Predict the GSK Composite Score [Range: 1-10] for
CC1=CC(=NC(=C1)C)C
Assistant: 7.708
Predict the GSK Composite Score [Range: 1-10] for ClCCCl.
Assistant: 4.304
```

In preliminary tests, we explored several prompt variations. When using common solvent names instead of SMILES strings in the descriptive prompt, we observed that the language models performed comparably; however, we believe this approach may not generalize well to novel solvents, as the model might not correctly interpret unknown names. In contrast, using solvent SMILES, which can be viewed as a chemical language, might allow the model to represent and predict properties for novel solvents more effectively. For further clarity, we explicitly integrated the numeric range of GSK scores (e.g., 1–10) to avoid the model producing text outputs and instead encourage outputs as float values. We also ensured that sufficient contextual information was provided, since the GSK score is not an intrinsic material property. These insights guided the final prompt design presented in the manuscript.

Regarding LLM model selection, we compared GPT-3.5-Turbo and GPT-4o for in-context learning, testing on 25 unseen cases, with the remaining data used as few-shot prompts, averaged over five repetitions. The average RMSE errors for GPT-3.5-Turbo and GPT-4o were 0.96 and 0.90, and the Pearson correlation coefficients were 0.683 and 0.688. The cost of in-context learning can be illustrated by the input and output token prices for these models: GPT-3.5 ($0.50/$1.50 per 1M tokens) is substantially lower than GPT-4o ($2.50/$10 per 1M tokens)[4]. Moreover, for fine-tuning, wherein additionally the number of models that can be run per day is also capped at a lower number for the newer models, GPT-3.5-Turbo ($8.00 per 1M tokens) is less than one-third the cost of GPT-4o models ($25.00 per 1M tokens)[5]. Since GPT-3.5-Turbo demonstrated comparable performance to GPT-4o, it was chosen in our study, further supported by cost considerations.

3 Model results summary

Three types of predictive workflows were considered for solvent sustainability prediction, quantified by the GSK SSG-based G-score as shown in the manuscript: the PolymRize™ Gaussian Process Regression (GPR) model, Fine-tuned GPT-3.5 Turbo models (FT GPT), and In-Context Learning with GPT-3.5 Turbo (ICL GPT). Additionally, similar models were evaluated for predicting the Environment, Health, Safety and Waste (EHSW) category scores, as illustrated in Figure 2.

$$\text{G-score} = \sqrt[4]{\text{Waste} \times \text{Environment} \times \text{Health} \times \text{Safety}} \tag{1}$$

Our results align with those in the manuscript: at small dataset sizes, language models perform slightly better, as GPR tends to predict nearly constant values. However, as the dataset size increases, all models converge to similar performance levels, with GPR consistently performing slightly better across all subcategory predictions.

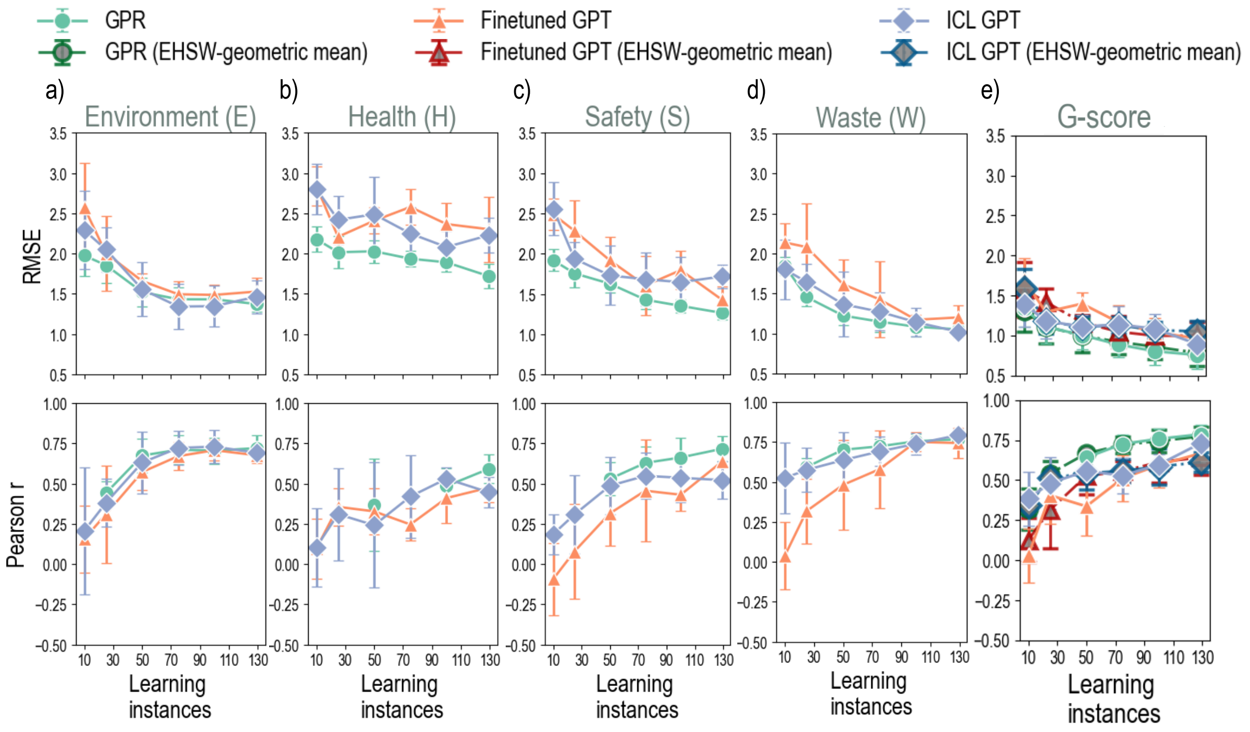

Figure 2: Model performance: Learning curves showing test set error metrics versus the number of learning instances — corresponding to training size for GPR and fine-tuned GPT models, and number of shots for in-context learning (ICL) — for (a) Environment, (b) Health, (c) Safety, (d) Waste categories, and (e) overall G-score. Error metrics are averaged over five runs using nested subsets and a fixed test set. Panel (e) additionally includes error metrics for G-scores predicted by dedicated G-score models as well as those calculated from the EHSW-based geometric mean of outputs from (a)–(d), shown as grey-filled markers, as described in Equation 1. No notable performance difference was observed between the dedicated G-score models and those calculated from the EHSW-based geometric mean. Although comparable, GPR consistently performs slightly better across EHSW and G-score models.

4 LOOCV GPR Outlier analysis

An analysis of outliers in the LOOCV GPR model for solvents was performed to better understand the model's limitations. For each prediction, we calculated the error as the difference between the predicted and target G-scores and then examined which points deviated from the average by more than about two standard deviations. As such, seven outliers were identified using this procedure and are numbered o1–o7. A closer inspection of these outliers reveals that each has a unique chemical profile that likely explains the difficulty of the ML model in generalizing predictions for these solvents.

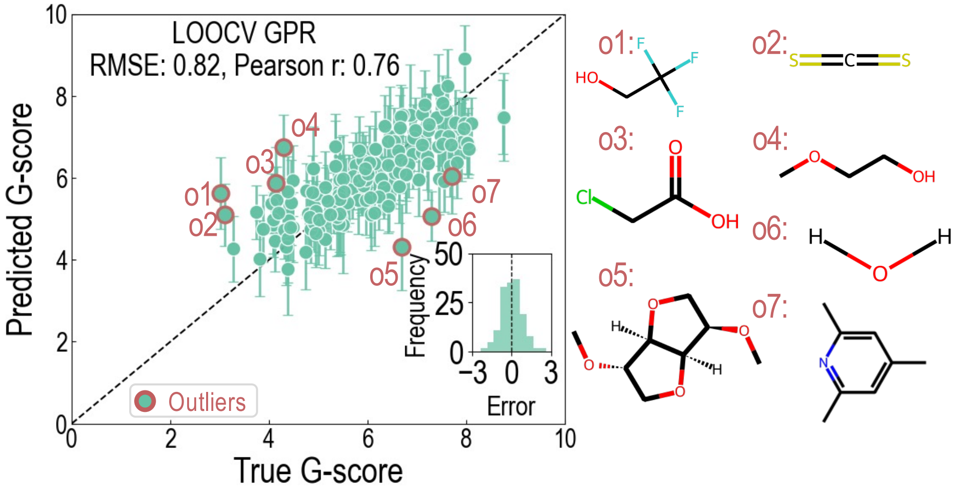

Figure 3: Outlier analysis (The figure is reproduced from the manuscript for ease of reference): (a) LOOCV parity plot for the best-in-class GPR model, with error bars representing prediction uncertainty and the seven identified outliers (o1–o7) are highlighted in red, with their chemical structures shown on the right.

For example, o1 corresponds to 2,2,2-trifluoroethanol, which contains a highly electron-withdrawing group that introduces unusually high polarity and strong hydrogen-bonding capacity, making it chemically distinct from other alcohols. Carbon disulfide (o2) is an outlier in the training data, as it is only one of four sulfur-containing molecules and the only sulfur-containing solvent without an oxygen or nitrogen atom. Moreover, o1 and o2 have the smallest G-scores, which may be affected by the limited representation of such molecules in the dataset. Similarly, chloroacetic acid (o3) has a strong electron-withdrawing chlorine substituent that significantly alters its dipolar character, making it chemically distinct from other acids. o4 (2-methoxyethanol) contains both an alcohol and an ether functional group, which may have influenced its prediction toward a higher G-score, as alcohols tend to be greener. o5 (dimethyl isosorbide) and o7 (2,4,6-collidine) are structurally very different from the majority of molecules in the training set. o6 (water) has an exceptionally high dielectric constant and significant hydrogen-bonding capabilities, making it difficult for the model features to fully capture its unique behavior.

5 G-score model predictions on recently discovered green solvents

Given that the GSK Solvent Selection Guide (SSG) was last updated in 2016, we reviewed green solvent literature published recently and identified a set of experimentally promising candidates not included in the original guide. This allowed us to test the applicability of our model to recently emerged solvents beyond the scope of the original SSG.

Table 1: Predicted G-scores and uncertainties for recently discovered green solvent candidates not included in the original GSK SSG.

**Solvent Name**	**Predicted G-score**	**Reference**
Eucalyptol	5.22 ± 1.00	[6,7]
Diethylene glycol ethyl ether	5.414 ± 0.79	[8]
Rose oxide	5.79 ± 1.13	[6]
Acetaldehyde diethyl acetal	5.87 ± 0.86	[6]
Pinene	6.02 ± 1.13	[6]
γ-terpinene	6.17 ± 1.13	[6]
Glycerol formal	6.32 ± 1.22	[9]
Ethyl levulinate	7.13 ± 0.86	[8]
Diethylene glycol dibenzoate	7.50 ± 0.97	[8]
Butyl levulinate	7.52 ± 0.97	[8]
Butyl lactate	7.18 ± 0.93	[8]
Diisobutyl succinate	7.94 ± 0.91	[8]
Diisobutyl glutarate	8.08 ± 0.95	[8]
Diisobutyl adipate	8.24 ± 0.95	[8]

It is clear that the model predicts the majority of these solvents to fall on the greener end of the spectrum, with all predicted G-scores exceeding 5.0 and several surpassing 7.0. Several bio-derived terpenes—such as rose oxide, pinene, γ-terpinene, and eucalyptol—exhibit moderate predicted G-scores, which aligns with the generally lower G-scores of aromatic compounds observed in the original GSK SSG. Notably, diisobutyl esters—including diisobutyl adipate, diisobutyl glutarate, and diisobutyl succinate—are predicted to have high G-scores. Literature supports the potential of these long-chain ester-based solvents as viable, greener alternatives to conventional petrochemical solvents, particularly in applications such as coatings and polymer dissolution.[8] Similarly, levulinates like ethyl levulinate and butyl levulinate also achieve high predicted G-scores, consistent with reports highlighting their promise as bio-based reaction media.[10]. Thus, this exercise highlights the applicability of the ML model to newly emergent green solvents.

References

(1) Byrne, F. P.; Jin, S.; Paggiola, G.; Petchey, T. H.; Clark, J. H.; Farmer, T. J.; Hunt, A. J.; Robert McElroy, C.; Sherwood, J. Tools and techniques for solvent selection: green solvent selection guides. *Sustainable chemical processes* **2016**, *4*, 1–24.

(2) Joshi, D. R.; Adhikari, N. An overview on common organic solvents and their toxicity. *J. Pharm. Res. Int* **2019**, *28*, 1–18.

(3) Browning, E. Toxic solvents: a review. *British Journal of Industrial Medicine* **1959**, *16*, 23.

(4) OpenAI Compare GPT-3.5 Turbo with Other Models. 2025; `https://platform.openai.com/docs/models/compare?model=gpt-3.5-turbo`, Accessed: 2025-10-01.

(5) OpenAI OpenAI API Pricing. 2025; `https://platform.openai.com/docs/pricing?ft-pricing=standard#fine-tuning`, Accessed: 2025-10-01.

(6) Gevorgyan, A.; Hopmann, K. H.; Bayer, A. Exploration of New Biomass-Derived Solvents: Application to Carboxylation Reactions. *ChemSusChem* **2020**, *13*, 2080–2088.

(7) Campos, J. F.; Scherrmann, M.-C.; Berteina-Raboin, S. Eucalyptol: a new solvent for the synthesis of heterocycles containing oxygen, sulfur and nitrogen. *Green Chemistry* **2019**, *21*, 1531–1539.

(8) Pilon, L.; Day, D.; Maslen, H.; Stevens, O. P.; Carslaw, N.; Shaw, D. R.; Sneddon, H. F. Development of a solvent sustainability guide for the paints and coatings industry. *Green Chemistry* **2024**, *26*, 9697–9711.

(9) Guerrero-León, L. A.; Bautista-Quijano, J. R.; Heffner, H.; Shilovskikh, V.; Campos, R.; Rivkin, B.; Vaynzof, Y. Novel Green Solvent for Sustainable Fabrication of Quasi-2D Perovskite Solar Cells. *Advanced Energy Materials* **2024**, *14*, 2402916.

(10) Adeleye, A. T.; Louis, H.; Akakuru, O. U.; Joseph, I.; Enudi, O. C.; Michael, D. P. A Review on the conversion of levulinic acid and its esters to various useful chemicals. *Aims Energy* **2019**, *7*, 165–185.
